# Case report: A rare case of desmoid-type fibromatosis originating in the small intestine

**DOI:** 10.3389/fmed.2023.1291945

**Published:** 2023-10-31

**Authors:** Junfeng Xie, Shichang Lai, Yangbiao Wang, Baolong Ye, Zhishun Huang, Kexing Xi

**Affiliations:** ^1^Department of Gastrointestinal Hernia Surgery, Ganzhou Hospital-Nanfang Hospital, Southern Medical University, Ganzhou, Jiangxi, China; ^2^Department of Thoracic Surgery, Ganzhou Cancer Hospital, Ganzhou, Jiangxi, China; ^3^Department of Emergency, The First Affiliated Hospital of Nanchang University, Nanchang, Jiangxi, China; ^4^Department of General Surgery, Nanfang Hospital, Southern Medical University, Guangzhou, China

**Keywords:** desmoid-type fibromatosis, small intestine, differential diagnosis, treatment, aggressive fibromatosis

## Abstract

**Background:**

Desmoid-type fibromatosis (DF) is characterized by a rare monoclonal fibroblast proliferation that exhibits variable and unpredictable clinical presentation. DF can be classified into sporadic and hereditary types. Despite extensive research efforts, the exact etiology of DF remains elusive.

**Case description:**

A 31-year-old male patient presented to the hospital with a progressively growing mass in the right lower abdomen, accompanied by abdominal discomfort. Symptoms are discovered 1 week before admission. Enteroscopy revealed no evidence of colonic abnormalities, and blood tests did not indicate any abnormalities. Due to the indeterminate nature of the mass during surgery, a partial resection of the ileum and cecum was performed, followed by ileocolonic end-to-end anastomosis, with no postoperative complications. The final pathological diagnosis confirmed primary desmoid-type fibromatosis of the distal ileum (invasive fibromatosis). To effectively manage DF, we recommend a follow-up schedule for patients. This includes appointments every 3 months in the first year following surgery, followed by appointments every 6 months up to the fifth year, and then once a year thereafter. The follow-up examinations should include collection of the patient’s medical history, physical examination, blood tests, ultrasounds, CT scans, and other relevant assessments. During the first year of the follow-up period, no further treatment was administered, and the patient remained disease-free.

**Conclusion:**

Desmoid-type fibromatosis (DF) originating from the small intestine is an extremely rare condition that exhibits local invasiveness and can be life-threatening. Despite its benign histology, DF has a high local recurrence rate and lacks metastatic potential. Diagnosis of DF remains challenging, especially in cases where surgical intervention is not feasible due to asymptomatic patients or partial organ impairment. In such cases, a “watchful waiting” approach is recommended as the initial treatment strategy. However, when preoperative diagnosis is difficult, surgery is typically considered the best option. Given the potential for local recurrence and the uncertain long-term prognosis, regular follow-up is necessary.

## Introduction

Desmoid-type fibromatosis (DF) is characterized by a rare monoclonal fibroblast proliferation that exhibits variable and unpredictable clinical behavior. Although DF is histologically classified as benign, it demonstrates local invasiveness and a high rate of local recurrence, without metastatic potential (1, 2). As per the World Health Organization (WHO) definition, DF is described as “a clonal fibroblast proliferation occurring in the deep soft tissues, characterized by infiltrative growth and a tendency to local recurrence, but not capable of metastasis” (3). While DF commonly affects the abdominal wall and trunk, its occurrence in the small intestine is extremely rare (2). Symptoms of DF can vary based on tumor location and size, often leading to confusion with other similar diseases.

This report presents a rare case of primary DF in the small intestine, highlighting clinicopathological features and differential diagnosis.

## Case report

**Figure 1:**
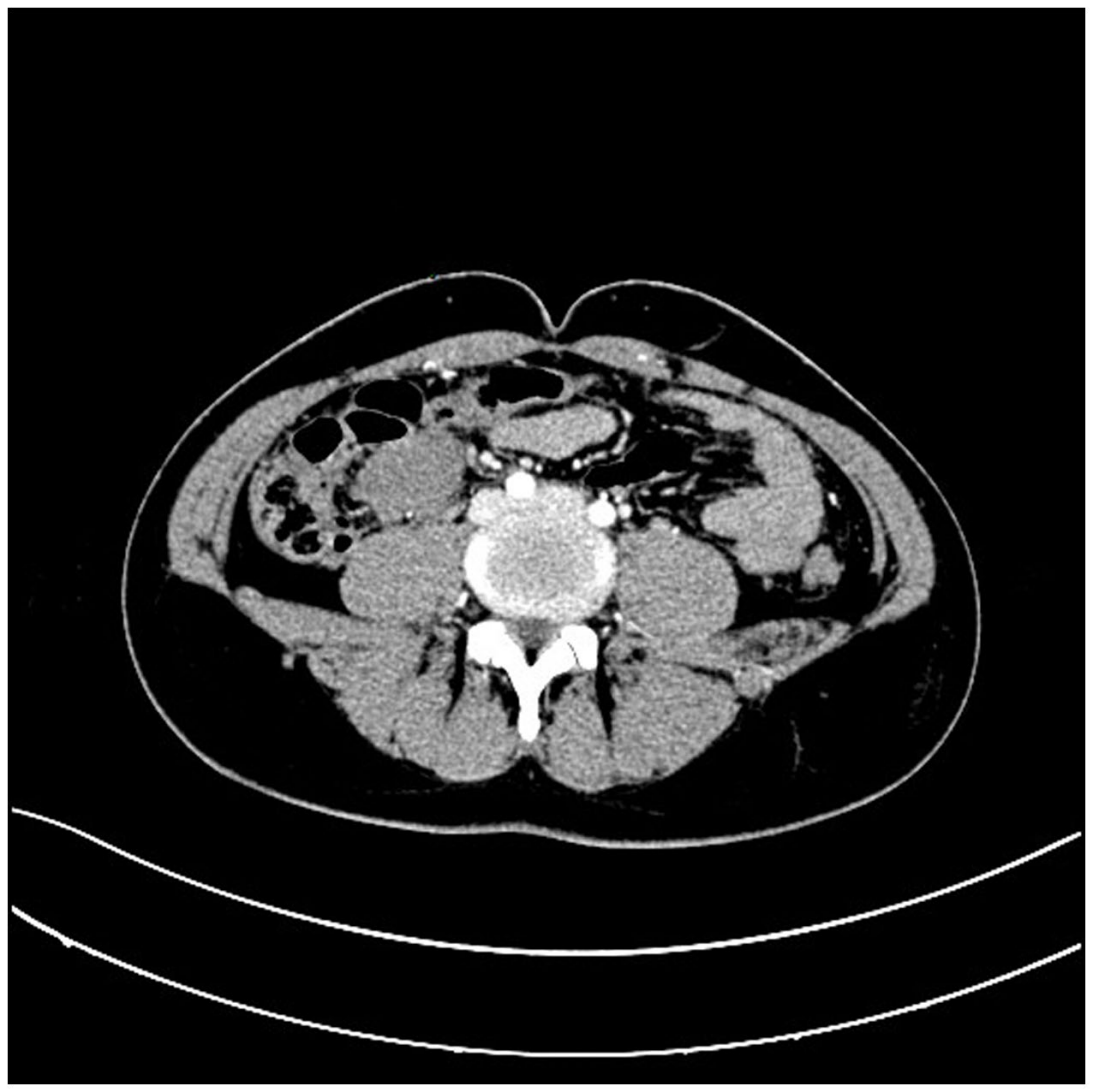
Abdominal CT scan revealed a well-defined mass in the right psoas muscle.

A 31-year-old male patient presented to the hospital with a progressively growing mass in the right lower abdomen, along with abdominal discomfort. Symptoms are discovered 1 week before admission. Prior to seeking care at our facility, the patient had undergone abdominal CT imaging at a community hospital, which revealed the presence of an abdominal mass. An abdominal CT scan revealed a well-defined mass measuring 3.9*2.9 cm, located anterior to the right psoas muscle at the level of the external iliac vessels (Figure 1). Enteroscopy revealed no evidence of colonic abnormalities, and blood tests did not indicate any abnormalities.

During exploratory laparotomy, the mass was identified in the distal ileum, exhibiting clear margins, firm consistency, and a maximum diameter of 4 cm. However, the nature of the mass could not be determined during surgery. Consequently, partial resection of the ileum and cecum was performed, followed by ileocolonic end-to-end anastomosis, with no postoperative complications.

**Figure 2:**
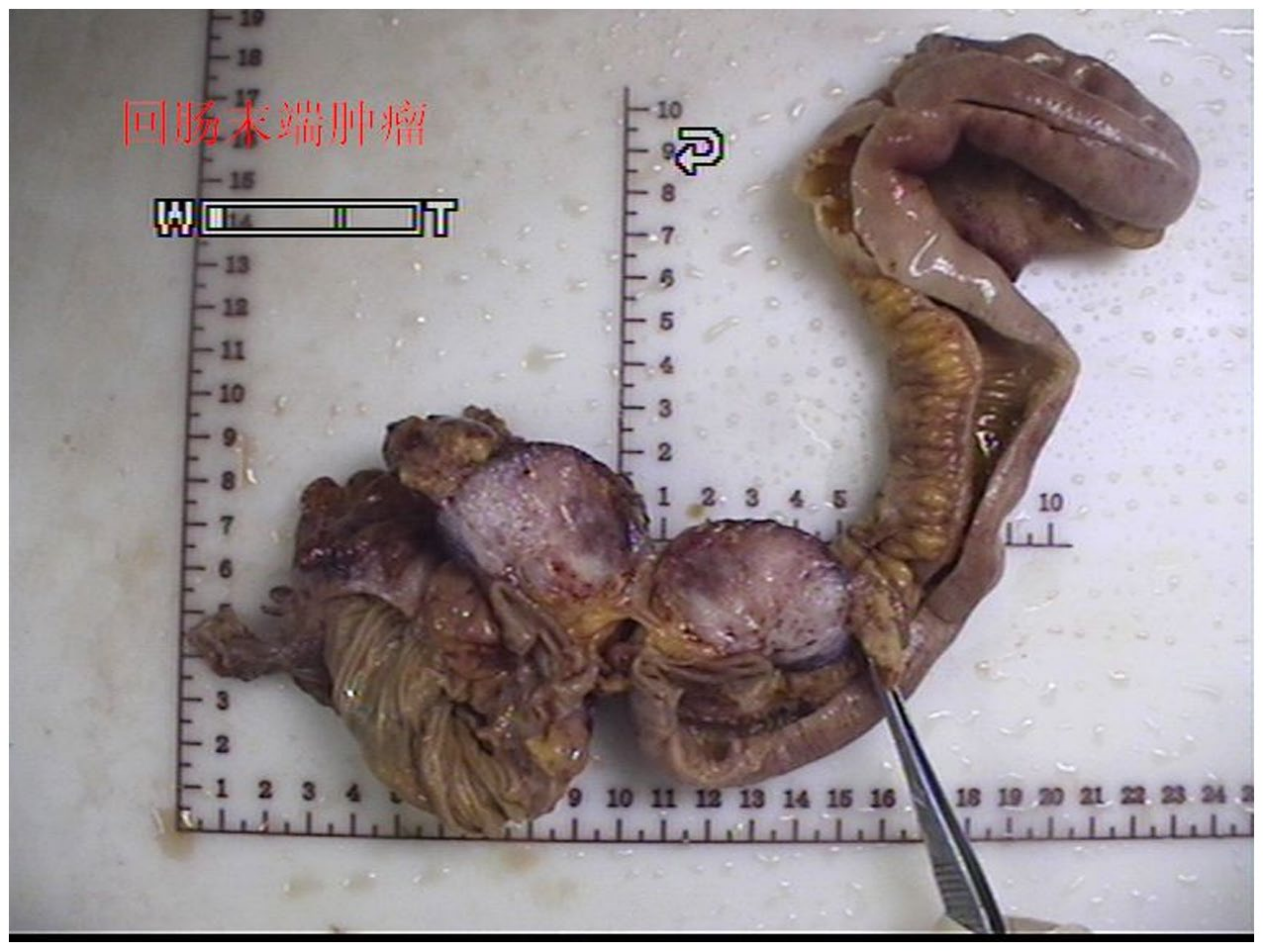
Gross examination showed a gray-white nodular mass with a firm and scar-like consistency.

**Figure 3:**
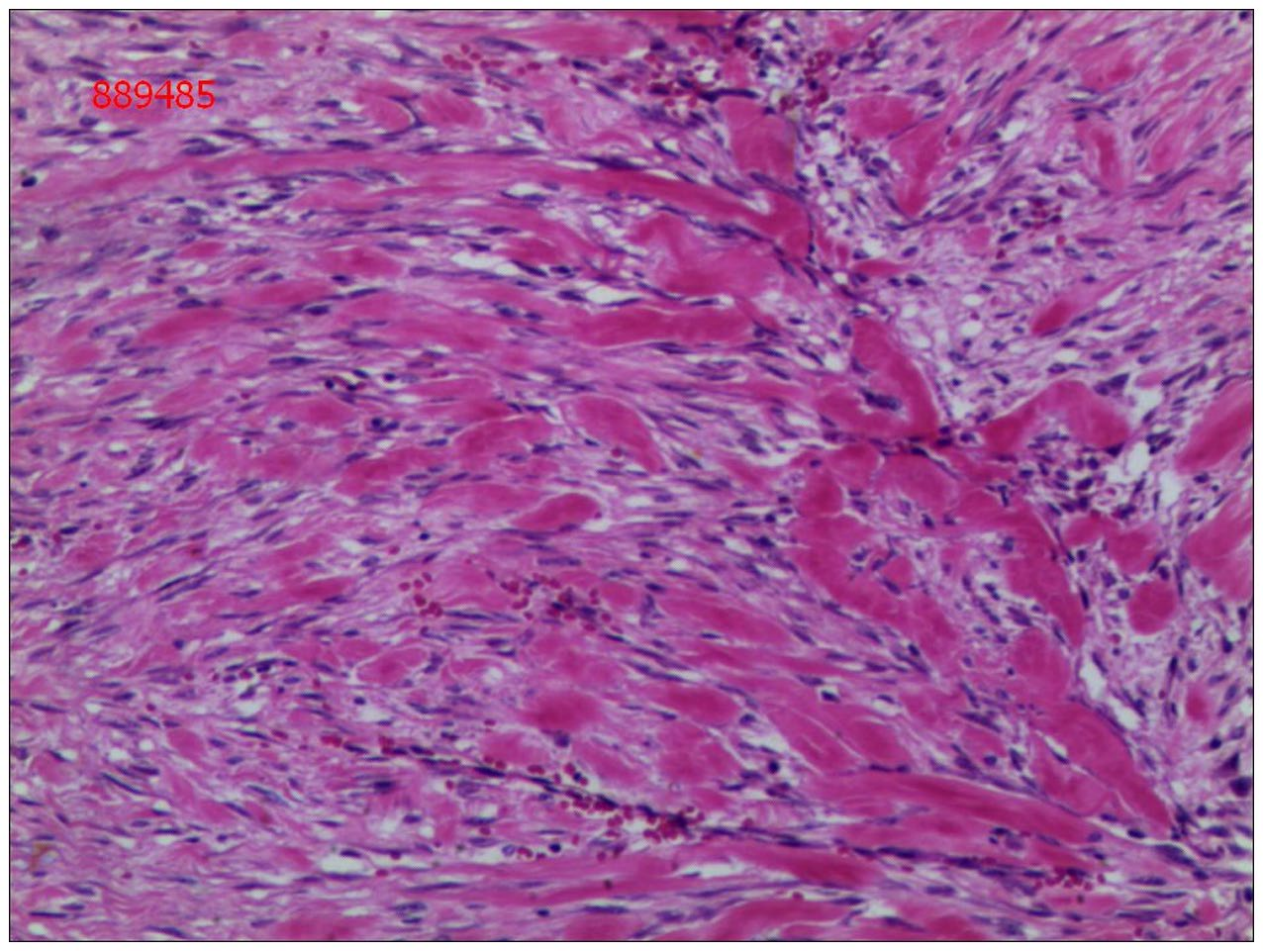
The cells were spindle-shaped, with abundant cytoplasm, and interspersed with collagen matrix (hematoxylin and eosin; magnification ×40).

**Figure 4:**
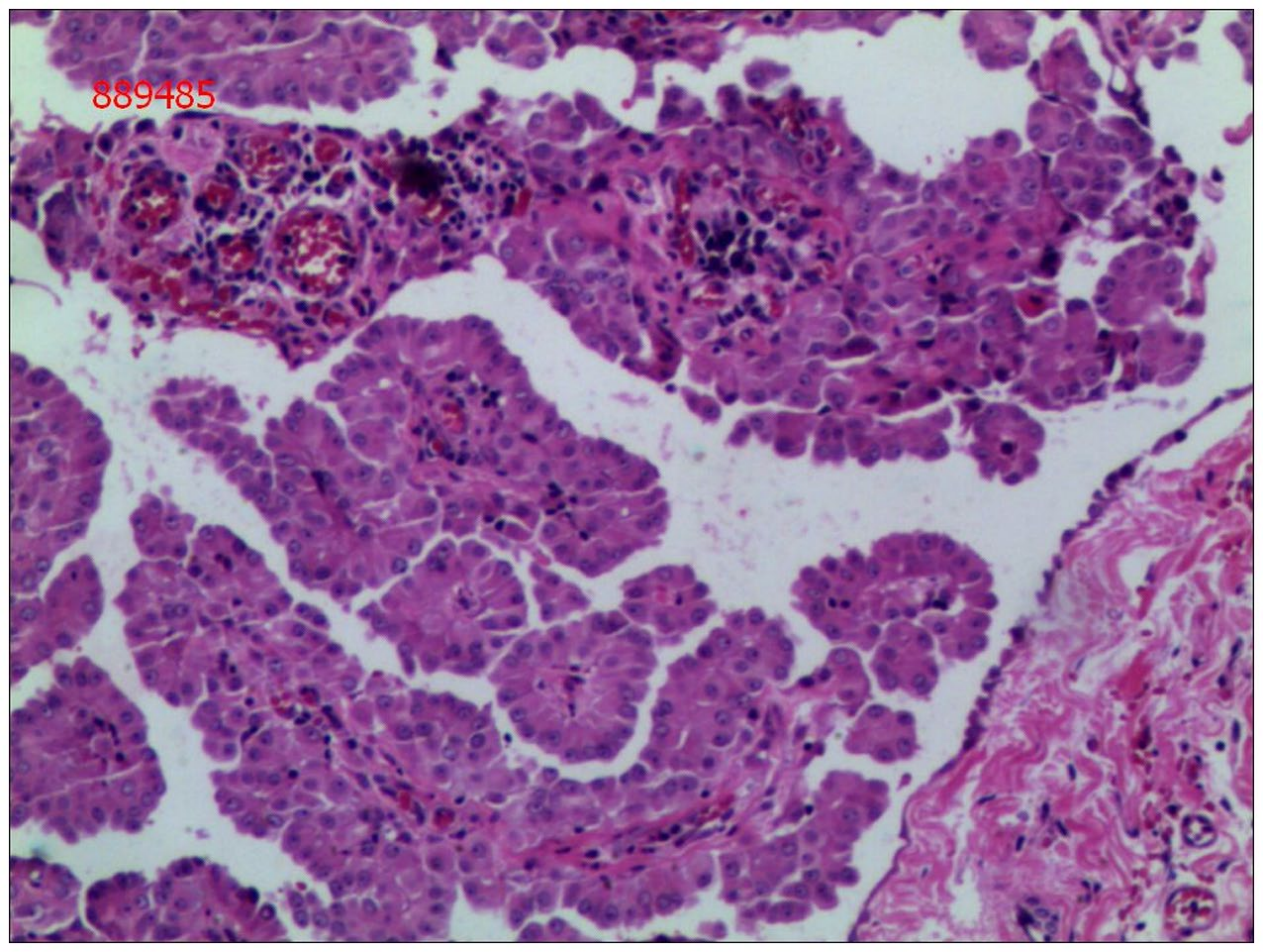
Spindle cells with interspersed collagen fibers(hematoxylin and eosin; magnification ×400).

Gross examination of the resected specimen revealed a grey-white nodular mass measuring approximately 4*3*5 cm, characterized by a firm and scar-like consistency (Figure 2). Histologically, the mass extended from the submucosal layer to the serosa of the distal ileum, infiltrating and growing within the intestinal wall and adipose tissue, displaying a fascicular or woven pattern arrangement. The spindle-shaped cells exhibited abundant cytoplasm, elongated nuclei with wavy features, interspersed with collagen fibers, and minimal infiltration of inflammatory cells (Figures 3, 4).

**Figure 5:**
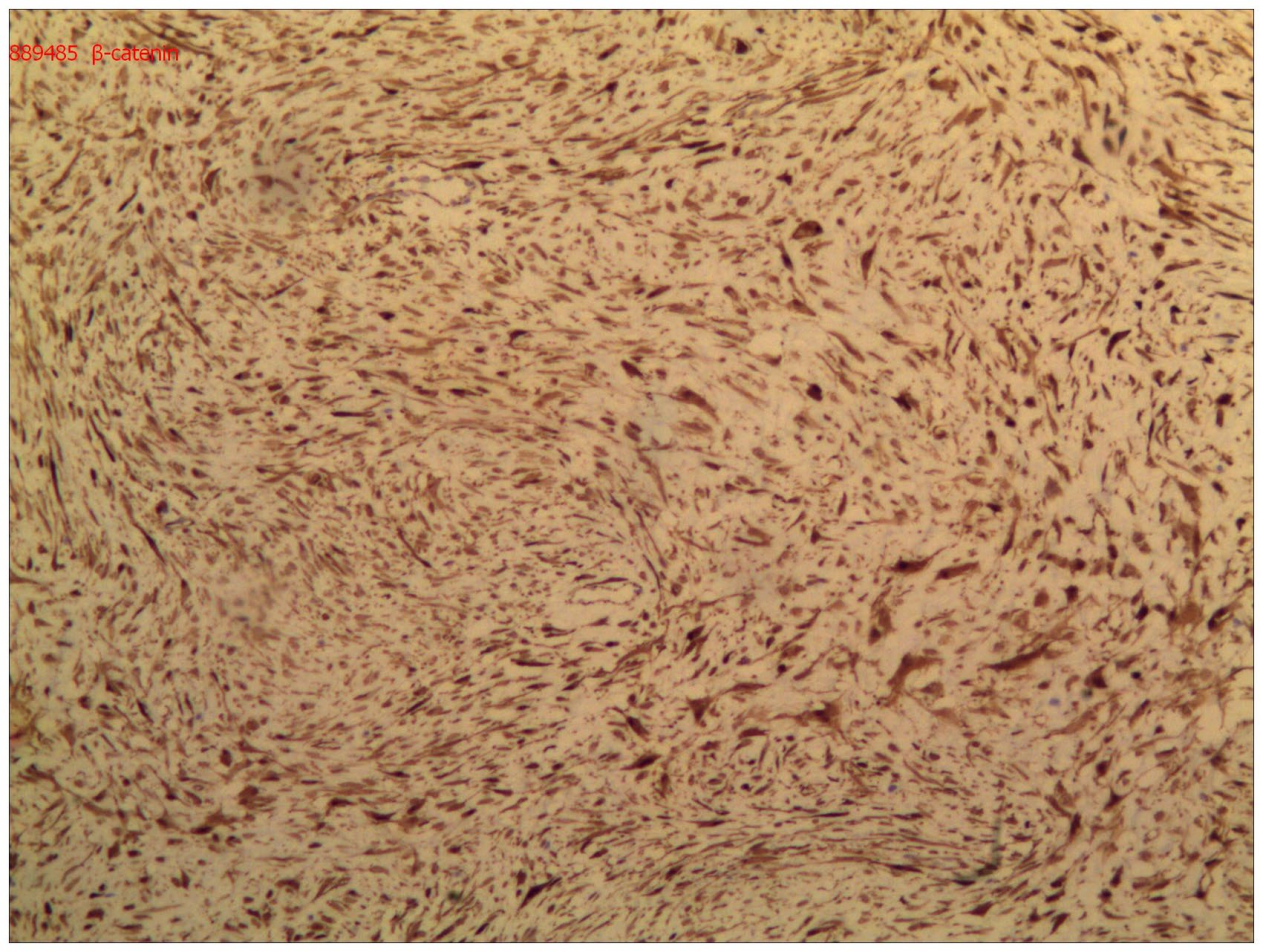
Immunohistochemical analysis demonstrated diffuse nuclear expression of β-catenin.

Immunohistochemical analysis demonstrated diffuse nuclear expression of β-catenin (Figure 5), along with nuclear positivity for vimentin (Vim), smooth muscle actin (SMA), and desmin (Des). The mass was negative for CD34, DOG-1, S-100, ENA, actin, and S-100. The final pathological diagnosis confirmed primary DF of the distal ileum (invasive fibromatosis).

Upon discharge from hospital, our patient had demonstrated clinical improvement and stability following surgery. Based on our findings, we recommend a regular follow-up schedule for patients. This includes appointments every 3 months during the first year post-surgery, followed by appointments every 6 months for the next 5 years, and then annual appointments thereafter. The follow-up examinations should encompass collection of medical history, physical examination, blood tests, ultrasounds, and CT scans, among other assessments. No additional treatment was administered after surgery, and the patient remained disease-free during the first year of follow-up.

## Discussion

Desmoid-type fibromatosis, also known as aggressive fibromatosis, exhibits diffuse and infiltrative growth into surrounding tissues without encapsulation histologically. The disease course is highly variable and unpredictable, encompassing growth, progression, stability, and even spontaneous regression (2, 4).

DF can be broadly categorized into two types: sporadic and hereditary DF. The sporadic form is considered a monoclonal disease originating from a single progenitor cell and typically affects the extra-abdominal or abdominal wall. The exact etiology of sporadic DF remains unknown, although previous studies have suggested associations with trauma (spontaneous or iatrogenic) and increased estrogen levels (3, 5–7). On the other hand, hereditary DF is more frequently observed in patients with familial adenomatous polyposis (FAP) and commonly presents as intra-abdominal DF in the mesentery and/or abdominal wall, with the mesentery being the preferred site in FAP-related cases (8, 9). FAP-related DF often demonstrates a more aggressive course, characterized by larger and more multifocal tumors occurring at an earlier stage compared to sporadic DF (2, 10). The primary occurrence of DF in the small intestine, as observed in our case, is exceptionally rare.

DF arises from proliferating myofibroblasts, and the Wnt/β-catenin pathway plays a central role in tumorigenesis (11). Most cases of DF exhibit mutations in the β-catenin gene. This mutation triggers a cascade of reactions that promote cell proliferation and differentiation, leading to DF development.

Clinically, the symptoms of DF vary depending on the tumor’s location, size, and growth rate. As the tumors typically display slow growth, many patients remain asymptomatic (12). Intra-abdominal tumors, such as the one in our case, may remain symptomless until they reach a larger size, causing abdominal discomfort or leading to the discovery of an abdominal mass. In severe cases, intestinal obstruction, ischemia, perforation, or bleeding may occur (13). The main symptom reported by the patient in our case was abdominal discomfort.

Macroscopically, DF appears as a firm, grayish-white, scar-like mass. It is characterized by heterogeneous, indistinct, and uniform proliferation of spindle-shaped cells, representing myofibroblasts surrounded by a collagen-rich matrix and lacking a capsule (4). Immunohistochemistry (IHC) assays show nuclear positivity for β-catenin, vimentin, Cox-2, c-KIT, PDGFRb, androgen receptor (AR), and estrogen receptor α (ERα) in DF. However, DF is negative for desmin, S-100, h-caldesmon, CD34, and c-KIT (2).

Ultrasound, CT, and MRI are the main diagnostic tools for DF, with CT being the preferred diagnostic and postoperative follow-up tool for intra-abdominal DF (14). The appearance of DF tissue varies depending on its composition (15). On CT scans, DF typically appears as an expansile, homogeneous or heterogeneously enhancing mass, often exhibiting calcifications (14). MRI images may show low, isointense, or high-intensity signals for DF (2). In our case, the identification of the small intestinal tumor was crucially aided by the abdominal CT scan.

Differential diagnosis of small intestinal DF includes tumors such as gastrointestinal stromal tumors (GIST), gastrointestinal clear cell sarcoma-like tumor (CCSLGT), gastrointestinal schwannoma, gastrointestinal leiomyoma, extranodal follicular dendritic cell sarcoma, and solitary fibrous tumor. Distinguishing DF from these tumors can be aided by the nuclear positivity of β-catenin in DF tumor cells and the CT/MRI scan images (16, 17).

The management of DF is complex and relies on individualized treatment approaches based on the tumor’s anatomical location and size. Various treatment modalities are available, including surgery, radiation therapy, and medical therapy. Previous studies have shown a lack of correlation between positive margins and local recurrence in DF. In some patients, tumors spontaneously stabilize or even disappear despite repeated recurrence (18, 19). In recent years, there has been a shift toward conservative non-operative strategies, particularly for asymptomatic patients or those with impaired partial organ function that can be diagnosed non-surgically. “Watchful waiting” is considered the first-line approach in such cases (3, 5, 20). However, diagnosing intestinal DF preoperatively is challenging, making surgery the recommended treatment option. Achieving R0 resection eliminates the need for postoperative adjuvant treatment. R1 or R2 resection does not automatically warrant a second resection; instead, priority is given to “watchful waiting.” If “watchful waiting” fails, surgical or medical therapy may be reconsidered (3, 5). For patients who decline surgical treatment, image-guided biopsies and rigorous observation should be considered. During the “watchful waiting” period, ultrasound monitoring is recommended for the first 2 months, followed by monitoring every 3 months in the first year, every 6 months until the fifth year, and subsequently annually. Implementing an intensive monitoring protocol, especially during the initial years, helps detect cases of rapid progression early (2, 21). Surgery remains the standard treatment method upon observation of tumor progression. In our case, R0 resection was achieved, and postoperative adjuvant therapy was not administered.

Due to the unique tumor location, radiation therapy is not suitable for intra-abdominal DF (22, 23). Medical therapy plays a significant role in managing progressive DF, especially when adjacent vital organs are involved or the lesions are unresectable (24). Options for medical treatment include hormone therapy, nonsteroidal anti-inflammatory drugs (NSAIDs), chemotherapy, and targeted therapy. Hormone-based treatment, using anti-estrogen agents like tamoxifen/toremifene alone or in combination with NSAIDs, is the first-line therapy for DF (8). Chemotherapy is typically employed as second-line treatment if hormonal therapy fails. Targeted therapy is primarily used when hormone therapy, NSAIDs, and chemotherapy have proven ineffective (25).

## Conclusion

In conclusion, DF originating from the small intestine is exceptionally rare. Despite its benign histology, it exhibits local invasiveness and potential life-threatening implications. Accurately distinguishing this lesion from other tumors is clinically significant for prognosis and treatment decisions. “Watchful waiting” remains the preferred first-line treatment approach for asymptomatic patients or those with impaired partial organ function that can be diagnosed non-surgically. Surgery is usually recommended when preoperative diagnosis is challenging. Upon achieving R0 resection, postoperative adjuvant treatment is not necessary. For R1 or R2 resections, routine second resection is not advised, prioritizing “watchful waiting.” Surgical or medical therapy may be reconsidered if “watchful waiting” fails. Due to the high risk of local recurrence and uncertain long-term prognosis, follow-up monitoring is essential.

## Data availability statement

The original contributions presented in the study are included in the article/supplementary material, further inquiries can be directed to the corresponding author.

## Ethics statement

Written informed consent was obtained from the individual for the publication of any potentially identifiable images or data included in this article.

## Author contributions

XJ: Conceptualization, Data curation, Formal analysis, Investigation, Methodology, Writing – original draft, Writing – review & editing. SL: Data curation, Investigation, Methodology, Writing – original draft, Writing – review & editing. YW: Data curation, Formal analysis, Investigation, Writing – original draft, Writing – review & editing. BY: Formal analysis, Investigation, Methodology, Writing – original draft, Writing – review & editing. ZH: Data curation, Investigation, Writing – original draft, Writing – review & editing. KX: Conceptualization, Data curation, Formal analysis, Investigation, Methodology, Project administration, Writing – original draft, Writing – review & editing.
